# DNA Profiling in Forensic Science: A Review

**DOI:** 10.1055/s-0041-1728689

**Published:** 2021-05-31

**Authors:** Jaya Lakshmi Bukyya, M L. Avinash Tejasvi, Anulekha Avinash, Chanchala H. P., Priyanka Talwade, Mohammed Malik Afroz, Archana Pokala, Praveen Kumar Neela, T K. Shyamilee, Vammi Srisha

**Affiliations:** 1 Department of Oral Medicine and Radiology, Tirumala Institute of Dental Sciences, Nizamabad, Telangana, India; 2 Department of Oral Medicine and Radiology, Kamineni Institute of Dental Sciences, Narketpally, Telangana, India; 3 Department of Prosthodontics, Kamineni Institute of Dental Sciences, Narketpally, Telangana, India; 4 Department of Pedodontics and Preventive Dentistry, JSS Dental College, Mysore, Karnataka, India; 5 Department of Oral Surgery and Diagnostic Sciences, Oral Medicine, College of Dentistry, Dar Al Uloom University, Riyadh, Kingdom of Saudi Arabia; 6 Department of Orthodontics, Kamineni Institute of Dental Sciences, Narketpally, Telangana, India; 7 Department of Oral Pathology, Private Practice, Hyderabad, Telangana, India; 8 Department of Oral Medicine and Radiology, Private Practice, Bangalore, Karnataka, India

**Keywords:** deoxyribonucleic acid, polymerase chain reaction, base pairs, single-nucleotide polymorphism, simple sequence repeat

## Abstract

DNA is present in most of the cells in our body, which is unique in each and every individual, and we leave a trail of it everywhere we go. This has become an advantage for forensic investigators who use DNA to draw conclusion in identification of victim and accused in crime scenes. This review described the use of genetic markers in forensic investigation and their limitations.

## Introduction

Forensic identification is a universal method used to establish the veracity in the process of forensic investigation. Both criminalities and medico-legal identification are integrative parts of forensic identification, having probative value. The value of an identification method resides in the specialist's ability to compare traces left at the crime scene with traces found on other materials such as reference evidence. Through this procedure, one can compare traces of blood, saliva, or any biological sample left at the crime scene with those found on a suspect's clothes and with samples from the victim. Medico-legal identification is based on scientific methods or intrinsic scientific methods absorbed from other sciences, usually bio-medical sciences. Scientific progress in the last 30 to 40 years has highlighted and continues to highlight the role of the specialists in identification. Their role proves its significance in cases that have to do with civil, family, and criminal law, as well as in cases of catastrophes with numerous victims (accidents, natural disasters, terrorist attacks, and wars). Together with the discovery by Mullis in 1983 of the polymerase chain reaction (PCR), Sir Alec Jeffreys in the field of forensic genetics used this technique by studying a set of DNA fragments that proved to have unique characteristics, which were nonrecurring and intrinsic for each individual, the only exception being identical twins. Alec Jeffreys named these reaction products “genetic fingerprints.” 1 PCR procedure is correct as per the reference.

## Brief History of Forensic Genetics

- In 1900, Karl Landsteiner distinguished the main blood groups and observed that individuals could be placed into different groups based on their blood type. This was the first step in development of forensic hemogenetics. 2
- 1915: Leone Lattes describes the use of ABO genotyping to resolve paternity case. 2
- 1931: Absorption–inhibition of ABO genotyping technique had been developed. Following on from this, various blood group markers and soluble blood serum protein markers were characterized. 2
- In the 1960s and 1970s: Developments in molecular biology, restriction of enzymes, Southern blotting, 3 and Sanger sequencing 4 enabled researchers to examine sequences of DNA.
- 1978: Detection of DNA polymorphisms using Southern blotting. 5
- 1980: First polymorphic locus was reported. 6
- 1983: A critical development in the history of forensic genetics came with the advent of PCR process that can amplify specific regions of DNA, which was conceptualized by Kary Mullis, a chemist; later he was awarded Nobel Prize in 1993. 7
- 1984: Alec Jeffrey introduced DNA fingerprinting in the field of forensic genetics, and proved that some regions in the DNA have repetitive sequences, which vary among individuals. Due to this discovery, first forensic case was solved using DNA analysis. 8

## DNA Structure and Genome

DNA was first described by Watson and Crick in 1953, as double-stranded molecule that adopts a helical arrangement. Each individual's genome contains a large amount of DNA that is a potential target for DNA profiling.

### DNA Structure

**Fig. 1:**
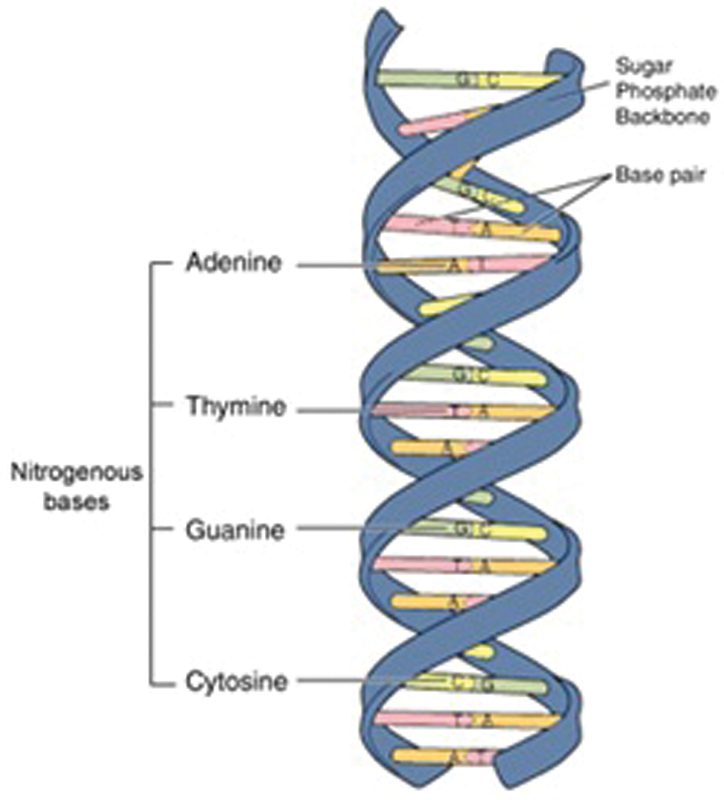
Structure of DNA. Image courtesy: National Human Genome Research Institute.

DNA is often described as the “blue print of life,” because it contains all the information that an organism requires in function and reproduction. The model of the double-helix structure of DNA was proposed by Watson and Crick. The DNA molecule is a polymer of nucleotides. Each nucleotide is composed of a nitrogenous base, a five-carbon sugar (deoxyribose), and a phosphate group. There are four nitrogenous bases in DNA, two purines (adenine and guanine) and two pyrimidines (cytosine and thymine). Each base is attracted to its complimentary base: adenine base always pairs with thymine base whereas cytosine base always pairs with guanine base ( Fig. 1 ). 9

## Organization of DNA into Chromosomes

There are two complete copies of the genome in each nucleated human cell. Humans contain ∼3,200,000,000 base pairs (BPs) of information, organized in 23 pairs of chromosomes. There are 2 sets of chromosomes; 1 version of each chromosome is inherited from each parent with total of 46 chromosomes. 10 11 12

### Classification of Human Genome 2

**Fig. 2:**
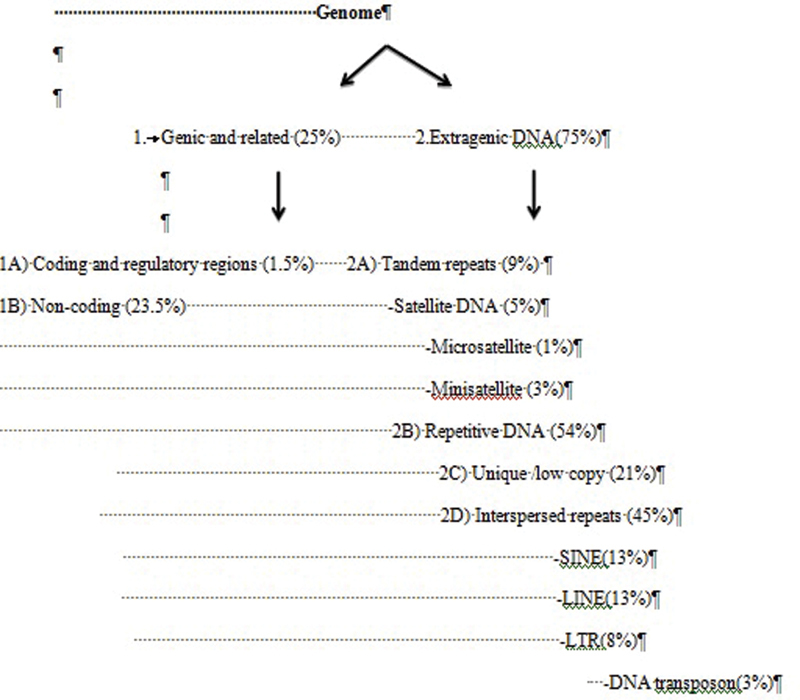
Classification of human genome.

Based on the structure and function, Classification of Human Genome into following different types ( Fig. 2 ).

1. **Coding and regulatory regions:** The regions of DNA that encode and regulate protein synthesis are called genes. Approximately, a human genome contains 20,000 to 25,000 genes; 1.5% of the genome is involved in encoding for proteins.
2. **Noncoding:** Overall, 23.5% of the genome is classified under genetic sequence but does not involve in enclosing for proteins; they are mainly involved with the regulation of genes including enhancers, promoters, repressors, and polyadenylation signals.
3. **Extragenic DNA:** Approximately 75% of the genome is extragenic, of which 50% is composed of repetitive DNA and 45% of interspersed repeats. Four common types of interspersed repetitive elements are: (i) short interspersed elements, (ii) long interspersed elements, (iii) long terminal repeats, and (iv) DNA transposons. Tandem repeats consist of three different types: (i) satellite DNA, (ii) minisatellite DNA, and (iii) microsatellite DNA.

### Genome and Forensic Genetics

DNA loci that are to be used for forensic genetics should have the following ideal properties:

- Should be highly polymorphic.
- Should be easy and cheap to characterize.
- Should be simple to interpret and easy to compare between laboratories.
- Should have a low mutation rate.

With recent advances in molecular biology techniques, it is possible to analyze any region with 3.2 billion BPs that make up the genome. 2

#### Biological Material

Three most important steps are collection, characterization, and storage.

#### Sources of Biological Evidence

Human body is composed of trillions of cells and most of them are nucleated cells, except for the red blood cells. Each nucleated cell contains two copies of individual's genome and can be used to generate a DNA profile. Usually, samples show some level of degradation but when the level of degradation is high, more cellular material is needed to produce a DNA profile. 13

Biological samples with nucleated cells are essential for forensic genetic profiling, such as: 14

- Liquid blood or dry deposits.
- Liquid saliva, semen, or dry deposits.
- Hard tissues like bone and teeth.
- Hair with follicles.

### Collection and Handling of Material at the Crime Scenes

Whole blood is considered as one of the widely used source of DNA. It is preserved in an anticoagulant (ethylenediamine tetra acetic acid) and conserved at 4°C for 5 to 7 days initially. After this period, DNA samples are kept at –20°C for few weeks or at –80°C for longer periods of time. Epithelial cells collected from crime scenes are harvested with sterile brush or bud. After harvesting, they are wrapped in plastic envelope or paper envelope and kept in a dry environment at room temperature. 15 It is essential that proper care is taken, such as maintaining integrity of the crime scene, wearing face masks and full protective suits during the investigation of scene, 16 17 18 as inappropriate handling of the evidence can lead to serious consequences. In worst cases, cross-contamination leads to high level of sample degradation; this can confuse or avert the final result of evidence.

## Characterization of DNA Analysis: Basic Steps 1

**Fig. 3:**
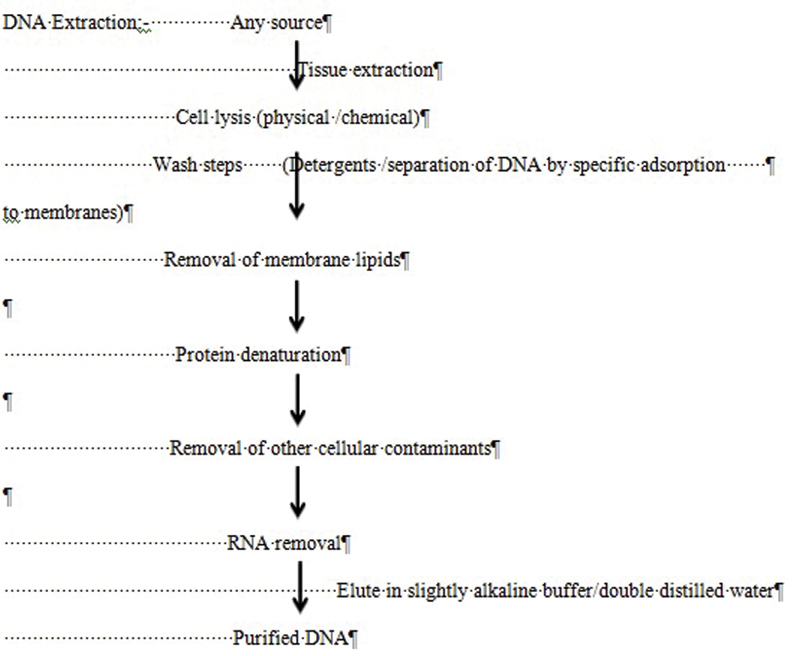
Extraction of DNA.

Analysis of DNA involves four basic steps, which are as follows ( Fig. 3 ):

1. DNA extraction.
2. DNA quantification.
3. DNA amplification.
4. Detection of the DNA-amplified products.

## DNA Extraction

The first DNA extraction was performed by Friedrich Miescher in 1869. Since then, scientists have made progress in designing various extraction methods that are easier, cost-effective, reliable, faster to perform, and producing a higher yield. With the advent of gene-editing and personalized medicine, there has been an increase in the demand for reliable and efficient DNA isolation methods that can yield adequate quantities of high-quality DNA with minimal impurities.

There are various methods of extraction as mentioned below, though commonly used are Chelex-100 method, silica-based DNA extraction, and phenol–chloroform method.

1. Chromatography-based DNA extraction method.
2. Ethidium bromide–cesium chloride (EtBr-CsCl) gradient centrifugation method.
3. Alkaline extraction method.
4. Silica matrices method.
5. Salting-out method.
6. Cetyltrimethylammonium bromide (CTAB) extraction method.
7. Phenol–chloroform method.
8. Sodium dodecyl sulfate (SDS)-proteinase K method.
9. Silica column-based DNA extraction method.
10. Magnetic beads method.
11. Cellulose-based paper method.
12. Chelex-100 extraction method.
13. Filter paper-based DNA extraction method.

### Chromatography-Based DNA Extraction Method

Chromatography-based DNA extraction method is used to isolate DNA from any kind of biological material. 19 This method is divided into three different types:

1. Size-inclusion chromatography: In this method, molecules are separated according to their molecular sizes and shape.
2. Ion-exchange chromatography (IEC): In this method, solution containing DNA anion-exchange resin selectively binds to DNA with its positively charged diethylaminoethyl cellulose group. 20 This method is simple to perform when compared with other DNA extraction methods. 19
3. Affinity chromatography: Protocol is similar to IEC; however it uses oligo that forms specific interaction with nucleic acid resulting in separation from the cell lysate. 19
  - This procedure is used for isolation of messenger ribonucleic acid (m-RNA).
  - It is time-efficient.
  - It yields a very good quality of nucleic acids. 21

### EtBr-CsCl Gradient Centrifugation Method

In 1957, Meselson et al developed this method. 22 DNA is mixed with CsCl solution, which is then ultra-centrifuged at high speed (10,000–12,000 rpm) for 10 hours, resulting in separation of DNA from remaining substances based on its density. EtBr is incorporated more into nonsupercoiled DNA than supercoiled DNA molecules resulting in accumulation of supercoiled DNA at lower density, and location of DNA is visualized under ultraviolet (UV) light.

**Advantage:**

- This method is used to extract DNA from bacteria.

**Limitations:**

- Greater amount of material source is needed.
- Time-consuming.
- Costly procedure due to long duration of high-speed ultra-centrifugation.
- Complicated method. 23

### Alkaline Extraction Method

First introduced by Birnboim and Doly in 1979, this method is used to extract plasmid DNA from cells. 24 Sample is suspended in NaOH solution and SDS detergent for lysis of cell membrane and protein denaturation. Potassium acetate is then added to neutralize the alkaline solution, which results in formation of precipitate. Plasmid DNA in the supernatant is recovered after centrifugation.

**Limitation:**

- Contamination of plasmid DNA with fragmented chromosomal DNA. 25

### Silica Matrices Method

The affinity between DNA and silicates was described by Vogelstein and Gillespie in 1979. 26

**Principle:** Selective binding of negatively charged DNA with silica surface is covered with positively charged ions. DNA tightly binds to silica matrix, and other cellular contaminants can be washed using distilled water or Tris-EDTA. 27

Advantages:

- Simple.
- Fast to perform.
- Cost-efficient.

**Limitation:**

- Silica matrices cannot be reused.

#### Salting-Out Method

Introduced by Miller et al 55 in 1988, this method is a nontoxic DNA extraction method.

**Procedure:** Sample is added to 3 mL of lysis buffer, SDS, and proteinase K, and incubated at 55 to 65°C overnight. Next, 6 mL of saturated NaCl is added and centrifuged at 2,500 rpm for 15 minutes. DNA containing supernatant is transferred into fresh tube and precipitated using ethanol. 28

**Advantages:**

- This method is used to extract DNA from blood, tissue homogenate, or suspension culture.
- High-quality DNA is obtained.
- Cost-efficient.
- Reagents are nontoxic.28,29

### Cetyltrimethylammonium Bromide (CTAB) Extraction Method

This method was introduced by Doyle et al in 1990. 30

Samples are added to 2% CTAB at alkaline pH. In a solution of low ionic strength, buffer precipitates DNA and acidic polysaccharides from remaining cellular components. Solutions with high salt concentrations are then added to remove DNA from acidic polysaccharides; later, DNA is purified using organic solvents, alcohols, phenols, and chloroform. 20

**Limitations:**

- Time-consuming method.
- Toxic reagents like phenol and chloroform are used.

### Phenol–Chloroform Method

This method was introduced by Barker et al in 1998. 31 Lysis containing SDS is added to cells to dissolve the cell membrane and nuclear envelope; phenol–chloroform–isoamyl alcohol reagent is added in the ratio 25:24:1. 28 Both SDS and phenol cause protein denaturation, while isoamyl alcohol prevents emulsification and hence facilitates DNA precipitation. The contents are then mixed to form biphasic emulsion that is later subjected to vortexing. This emulsion separates into two phases upon centrifugation, upper aqueous phase, composed of DNA, and the lower organic phase, composed of proteins. Upper aqueous phase is transferred to fresh tube and the lower organic phase is discarded. These steps are further repeated until the interface between the organic and aqueous phase is free from protein. 31 Later, sodium acetate solution and ethanol are added in 2:1 or 1:1 ratio, followed by centrifugation for separation of DNA from the solution. The pellet is washed with 70% ethanol to remove excess salt from the DNA and subjected to centrifugation for removal of ethanol. The pellet is dried and suspended in an aqueous buffer or sterile distilled water.

**Advantages:**

- Used to extract DNA from blood, tissue homogenate, and suspension culture.
- Inexpensive.
- Gold standard method.

**Limitation:**

- Toxic nature of phenol and chloroform. 28

#### SDS-Proteinase K Method

It was first introduced by Ebeling et al in 1974. 32 For extraction of DNA, 20 to 50 µL of 10 to 20 mg/mL proteinase K is added. SDS is added to dissolve the cell membrane, nuclear envelope, and also to denature proteins. The solution is incubated for 1 to 18 hours at 50 to 60°C and then DNA can be extracted using the salting-out method or phenol–chloroform method. 33

#### Silica Column-Based DNA Extraction Method

In this method, 1% SDS, lysis buffer (3 mL of 0.2 M tris and 0.05 M EDTA), and 100 mg of proteinase K are added to sample and incubated at 60°C for 1 hour, and this mixture is added in a tube containing silica gel. To this, phenol–chloroform is added in the ratio of 1:1 and centrifuged for 5 minutes. This separates the organic phase containing proteins beneath the silica column while aqueous phase containing DNA above the gel polymerase, and then aqueous layer is transferred to the tube and dissolved in TE buffer.

**Advantages:**

- Increase in purity of extracted DNA.
- Silica gel prevents physical contact with toxic reagents.
- DNA yield is 40% higher than organic solvent-based DNA extraction method.34

### Magnetic Beads Method

Trevor Hawkins filed a patent “DNA purification and isolation using magnetic particles” in 1998. 35

Magnetic nanoparticles are coated with DNA-binding antibody or polymer that has specific affinity to bind to its surface. 36 In this method, a magnetic field is created at the bottom of the tube using an external magnet that causes separation of DNA-bound magnetic beads from cell lysate. The supernatant formed is rinsed, and beads aggregated at the bottom can be eluted with ethanol precipitation method; and the magnetic pellet is incubated at 65°C to elute the magnetic particles from the DNA. 28

**Advantages:**

- Time taken is less than 15 minutes.
- Faster compared with other conventional methods.
- Little equipment is required.
- Less cost.19,37

### Cellulose-Based Paper

It was first introduced by Whatman in 2000, who filed a patent titled “FTA-coated media for use as a molecular diagnostic tool.” Cellulose is a hydroxylated polymer with high binding affinity for DNA. Whatman FTA cards are commercially available as cellulose-based paper that is widely used for extraction of DNA. 38 They are impregnated with detergents, buffers, and chelating agents that facilitate DNA extraction. About 1 to 2 mm of sample area is removed with micro punch and further processed for downstream applications. 19 39

**Advantages:**

- Extraction of DNA using cellulose-based paper is fast.
- Highly convenient.
- Does not require laboratory expertise.
- Easy storage of sample.40

### Chelex-100 Extraction Method

In 2011, Xlonghui et al 40 patented a DNA extraction method using Chelex-100. Chelex resin is used to chelate metal ions acting as cofactors for DNases. After incubating overnight, 5% Chelex solution and proteinase K are used to degrade the added DNases, which are later boiled in 5% Chelex solution to lyse the remaining cell membranes, and to denature both proteins and DNA. Also, 5% Chelex solution prevents DNA from being digested by DNases that remain after boiling, hence stabilizing the preparation. The resulting DNA can then be concentrated from the supernatant after centrifugation.

**Advantages:**

- Reduced risk of contamination.
- Use of single test tube.

**Limitation:**

- Isolated DNA can be unstable. 38

### Filter Paper-Based DNA Extraction Method

This method was described by Ruishi and Dilippanthe in 2017. DNA extraction method using filter paper can be used to isolate DNA from plant sources. A spin plate composed of 96-well plate is used, with a hole 1 mm in diameter drilled into bottom of each well used, and each well containing a disk of 8 mm diameter Whatman FTA filter paper. Samples subjected to lysis buffer are filtered with centrifugation.

**Advantage:**

- Less cost. 41

## DNA Quantification

After DNA extraction, an accurate measurement of the amount and quality of DNA extract is desirable. When the correct amount of DNA is added to PCR, it results in best quality within short duration of time. Adding less or more amount of DNA will results in a profile that is difficult or impossible to interpret. 40

Quantity of DNA that can be extracted from a sample depends on the type of model. Quantity of DNA in different biological samples is shown in Table 1 . 42

**Table 1:** Various sources of biological evidence

Type of sample	Amount of DNA
Liquid blood	30,000 ng/mL
Stain of blood	200 ng/cm ^2^
Liquid saliva	5,000 ng/mL
Hair (with root) shed	1–12 ng/root
Hair (with root) plucked	1–750 ng/root
Liquid semen	250,000 ng/mL
Postcoital vaginal swab	0–3,000 ng/swab
Oral swab	100–1,500 ng/swab
Urine	1–20 ng/mL
Bone	3–10 ng/mg
Tissue	50–500 ng/mg

### Classification of Quantification 43

DNA quantification can be classified as follows:

1. Nonnucleic acid-based quantification methods.
  - Microscopic and macroscopic examination.
  - Chemical and immunological methods.
2. DNA-based total genomic methods.
  - Intact and degraded DNA–UV spectrometry.
    ○ PicoGreen homogenous microtiter plate assays.
  - Intact vs degraded DNA–agarose gel electrophoresis.
3. Real-time PCR, DNA-based target specific methods.
  - Human total autosomal DNA.
  - Y chromosome DNA, mitochondrial DNA (mt-DNA), Alu repeat real-time PCR.
  - Multiplex real-time PCR.
4. End-point PCR DNA quantification and alternative DNA detection methods.
5. RNA-based quantification.

### Visualization on agarose gels

**Advantages:**

- It is relatively easy and quick method for assessing both quality and quantity of extracted DNA.
- Gives indication of size of extracted DNA molecules.

**Disadvantages:**

- Quantification is subjective.
- Total DNA obtained can be mixture of human DNA and microbial DNA and this can lead to overestimation of DNA concentration. 2

### Ultraviolet Spectrometry

Spectrometry is commonly used for quantification of DNA in molecular biology but has not been widely adopted by the forensic community. Usually, DNA absorbs light maximally at 260 nm; this feature is used to estimate the amount of DNA extraction by measuring wavelengths ranging from 220 nm to 300 nm. With this method, it is possible to assess the amount of protein (maximum absorbance is 280 nm) and carbohydrate (maximum absorbance is 230 nm). If the DNA extract is clean, the ratio of absorbance should be between 1.8 and 2.0.

**Disadvantages:**

- Difficult to quantify small amounts of DNA.
- It is not human specific. 2

### Fluorescence Spectrometry

EtBr or 4′,6 diamidino-2-phenylindole can be used to visualize DNA in agarose gels. In addition to staining agarose gels, fluorescent dyes can be used as an alternative to UV spectrometry for DNA quantification. PicoGreen dye is commonly used because it is specific for double-stranded DNA as it has the ability to detect little amount of DNA as 25 pg/mL.

**Disadvantage:**

- Nonhuman specific. 44

## DNA Amplification

There are eight DNA- and RNA-based techniques, but PCR and reverse transcription-PCR have been the predominant techniques.

PCR is the commonly used method of amplification of DNA. PCR amplifies specific regions of DNA template; even a single molecule can be amplified to 1 billion fold by 30 cycles of amplification. 45

DNA amplification occurs in cycling phase, which consists of three stages.

1. Denaturation.
2. Annealing.
3. Extraction.

Normal range of PCR cycle is between 28 and 32; when DNA is very low, then cycles can be increased to 34 cycles. 46

Other methods are as follows: 47

- Nucleic acid sequence-based amplification method.
- Strand displacement amplification.
- Recombinase polymerase amplification.
- Strand invasion-based amplification.
- Multiple displacement amplification.
- Hybridization chain reaction.

After the amplification of DNA, the final step is detection of the DNA-amplified products.

## Detection of the DNA-Amplified Products

The following methods are used in forensic human identification:

1. Autosomal short-tandem repeat (STR) profiling
2. Analysis of the Y chromosome
3. Analysis of mt-DNA.
4. Autosomal single-nucleotide polymorphism (SNP) typing.

Autosomal STR Profiling

STRs were discovered in 1980. Since then, they are considered as gold standard in human identification in forensics. STR or microsatellites are the most frequently genotyped to distinguish between individuals. STR consists of mononucleotide, dinucleotide, trinucleotide, tetranucleotide, pentanucleotide, and hexanucleotide repeats of which tetranucleotide repeats are used for genotyping. 2

STR profiling is used in paternity/maternity testing, rape perpetrators' identification, kinship testing, and disaster victim identification. 48

STR-based DNA analysis in forensic has been well accepted by professionals and population as an important tool in criminal justice and in human identification.

**Advantages:**

- The test is simple.
- Can be done rapidly. 49

### Analysis of the Y Chromosome

Typically, biologically a male individual has 1 Y chromosome and contains 55 genes. Because of this unique feature, analysis of Y chromosome is done in crime cases. 50

**Application of Y chromosome in forensic medicine:** It is present only in males. Thus, in crime cases, the investigators expect to find Y chromosome at the crime scene. Also, when talking about male–female ratio in body fluid mixtures, such as sexual assault or rapes, by analyzing the Y-STR component, the investigators can obtain more information regarding the male component. It is well known that azoospermic or vasectomized rapists do not leave semen traces, and it is impossible to find spermatozoa on the microscopic examination. In such cases, the Y-STR profiling is very useful, offering information regarding the identity of the accused person. 50

### Analysis of Mitochondrial DNA (mt-DNA)

mt-DNA is inherited from mother; thus all the members of a matrilineal family share the identical haplotype.

**Advantages:**

- mt-DNA has 200 to 1,700 copies per cell.
- Increased probability of survival when compared to nuclear DNA.

**Applications:**

- Analysis of biologic samples that are severely degraded or old.
- Samples with low amount of DNA (e.g., hair shafts). 51

### Autosomal Single-Nucleotide Polymorphism Typing

SNP has a lower heterozygosity when compared with STRs. Advantage of SNP typing over STR is that the DNA template size can be as large as 50 BPs, compared with STRs that need a size of 300 BPs to obtain good STR profiling. 52 Due to this reason, SNP has become an important tool in analyzing degraded samples. Thus in the 2001 World Trade Center disaster, victims were identified using SNP typing. 53 54

### Impact of Genetic Identification in Justice 1

Genetic testing using DNA has been widely applicable to the field of justice. This method is being used for the following:

- Identification of accused and confirmation of guilt.
- Exculpation of innocent ones.
- Identification of persons who commit crimes or serial killers.
- Identification of victims in disasters.
- Establishing consanguinity in complex cases.

## Conclusion

Currently, the DNA genotyping of all types of microtraces or biological traces containing nucleated cells is possible if they are not entirely demolished, either chemically or by bacteria. The DNA analysis is an important tool in solving caseworks in forensic medicine, such as establishing the custody of a child through paternity or maternity tests, identifying victims from crimes or disasters, or exonerating innocent people convicted to prison.
